# Knowledge and beliefs concerning evidence-based practice amongst complementary and alternative medicine health care practitioners and allied health care professionals: A questionnaire survey

**DOI:** 10.1186/1472-6882-8-45

**Published:** 2008-07-23

**Authors:** Julie Hadley, Ismail Hassan, Khalid S Khan

**Affiliations:** 1 The Education Resource Centre. Birmingham Women's Health Care NHS Trust, Edgbaston, Birmingham, B15 2TG, UK; 2 The University of Birmingham, Edgbaston, Birmingham, B15 2TT, UK

## Abstract

**Background:**

Evidence-based practice (EBP) has become an important competency in many allied and complementary and alternative medicine (CAM) health care practitioners' professional standards of proficiency.

**Methods:**

To compliment an EBP course for allied health care professionals and CAM practitioners, we undertook a questionnaire survey to assess learning needs. We developed a questionnaire to measure allied health care professionals and CAM practitioners' basic knowledge, skills and beliefs concerning the main principles of EBP. The questionnaires were administered to all attendees of one-day EBP workshops.

**Results:**

During 2004–5 we surveyed 193 allied health care professionals and CAM practitioners who attended one-day EBP courses prior to commencement of teaching. Of the respondents 121 (62.7%) were allied health care professionals and 65 (33.7%) practitioners stated that they work in the CAM field Our survey found that the majority of the respondents had not previously attended a literature appraisal skills workshop (87.3%) or received formal training in research methods (69.9%), epidemiology (91.2%) or statistics (80.8%). Furthermore, 67.1% of practitioners specified that they felt that they had not had adequate training in EBM and they identified that they needed more training and education in the principles of EBM (86.7%). Differences in knowledge and beliefs concerning EBP amongst allied and CAM practitioners were found and length of time since qualification was also found to be an important factor in practitioner's beliefs. More CAM practitioners compared to allied health professionals accessed educational literature via the Internet (95.3% v 68.1%, p = 0.008). Whilst, practitioners with more than 11 years experience felt that original research papers were far more confusing (p = 0.02) than their less experienced colleagues.

**Conclusion:**

The results demonstrate that practitioner's learning needs do vary according to the type of profession, time since graduation and prior research experience. Our survey findings are exploratory and will benefit from further replication, however, we do believe that they warrant consideration by allied health care and CAM tutors and trainers when planning EBP teaching curricula as it is important to tailor teaching to meet the needs of specific subgroups of trainees to ensure that specific learning needs are met.

## Background

Evidence-Based Practice (EBP) requires that decisions about health care are based on the best available, current, valid and relevant evidence. These decisions should be made by those receiving care, informed by the tacit and explicit knowledge of those providing care [1]. Regardless of speciality, teaching of evidence-based medicine (EBM) or EBP has become part of the core medical training in the United Kingdom [2] and has also become recognised as an important competency in many allied [3-10] and complementary (CAM) [11-13] health care practitioners' professional standards of proficiency. Furthermore, a report from the UK House of Lords [14] has recommended that that every therapist working in CAM should have a clear understanding of the principles of evidence-based healthcare.

The opportunities for CAM practitioners to learn the principles of EBP are limited. We found no courses available in our region. As both allied health care professionals and CAM practitioners are not catered for in traditional EBM postgraduate medical training, we developed an EBP course for allied health care professionals and CAM practitioners. The course was designed so that it had a core curriculum, which could be adapted specifically to the individual needs of the students [15]. To ensure that relevant teaching and learning opportunities were realised, a needs assessment exercise was undertaken, as recommended by Harden [16] as an essential criteria which should be carried out when designing and developing any course and particularly because EBP has not been a mandatory requirement of their training until recently. The findings of such an exercise can provide critical evidence for development and tailoring of EBP curricula improving the effectiveness of teaching. We undertook a needs assessment exercise using a questionnaire survey of allied health care professionals and CAM practitioners' knowledge, skills and beliefs regarding EBP in the West Midlands region. This allowed us the opportunity to compare and contrast different needs of groups according to specialty and time since qualification and to tailor our course to meet their specific needs

## Methods

During 2004–5 we surveyed 193 allied health care professionals and CAM practitioners who attended one-day EBP courses prior to commencement of teaching. Allied health care professionals included Physiotherapists, Chiropodists, Dentists, Nurses, Midwives, Sport injuries specialists, Nursing assistants and Pharmacists. CAM practitioners included Osteopaths, Chiropractors, Alexander technique practitioners, Acupuncturists, Herbalists, Feng Chui practitioners, Homoeopaths, Hypnotherapists, Shiatsu practitioners, and Reflexologists. Invitations were sent out to all CAM practitioners and allied health care professionals listed in a database of all registered practitioners that we developed by interrogating the West Midlands regional directory and relevant professional associations. In total 859, CAM practitioners and allied health care professionals were approached and 193 (22.5%) completed the questionnaire. Attendance to these courses was free and not mandatory. The courses were funded from a grant received from the Learning and Skills Council and European Social Fund (European Union Grant LSE31068WM2). The study was planned prospectively using recommended methods for educational needs analyses[16] and questionnaire surveys [17]. Ethical approval for the study was not required. Participants were made aware of the purpose of the survey, the anonymous nature of the dataset generated and the option to not respond if they so wished. This information served as the basis for an informed consent from each respondent.

We developed a questionnaire to measure allied health care professionals and CAM practitioners' basic knowledge, skills and beliefs concerning the main principles of EBP including questions from previously published and validated questionnaires [18-21]. The questionnaire included questions relating to the practitioners' self assessment of their literature searching behaviour, their self perceived knowledge of their own critical appraisal skills and beliefs. Multiple choice answers and six-point Likert scales were used to measure responses, without a 'don't know' or neutral point on the scale. However, participants were instructed to tick a box if they did not understand the question. Questions about knowledge included statements relating to how confident the respondents feel about assessing research methodology. The statements address perceived self-confidence in interpreting statistical tests, evaluating bias and assessing sample size. Answers were scored from '1' not confident at all to '6' very confident. Items on beliefs about EBP included statements such as 'EBP is essential in my practice', 'clinical judgement is more important than EBP' and 'I feel that I need more training in EBP'. Participants scored their answers on a range from '1–6', with '1' indicating that they disagreed strongly with the statement and '6' suggesting that they agreed strongly with the statement [see Additional file 1].

The questionnaires were self-administered by the candidates on arrival to the teaching session. All data obtained were entered into a Microsoft Excel spreadsheet and exported for analyses using SPSS software version 14.0 (SPSS Inc., Chicago, IL., USA). The data then was coded and participants categorised into groups according to their background. Descriptive statistics were computed when possible. The data was summarized as counts (or percentages) occurring in the various response categories. Paired Likert-type items or sets of items were compared using nonparametric statistical techniques (e.g. chi-square homogeneity tests, Mann-Whitney-Wilcoxon U test) [22]. Differences on categorical measures were reported as P value. The result was significant if P < 0.05. We compared responses from allied health care professionals with those from CAM practitioners.

## Results

In total, 193 allied health care professionals and CAM practitioners completed the questionnaire. Of the respondents 121 (62.7%) were allied health care professionals and 65 (33.7%) practitioners stated that they work in the CAM field (7 missing responses). Furthermore, 91 (47.1%) had qualified or had been working in their chosen health care fields within the last 10 years, whilst 82 (42.5%) had been qualified or working for 11 years and over.

**Figure 1:**
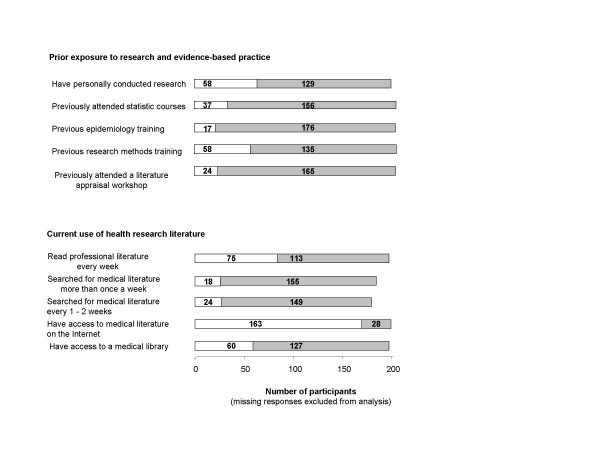
Background of allied health care professionals and CAM practitioners' prior exposure to research and evidence-based practice and current use of health research literature.

Figure 1 illustrates the respondents' background of exposure to research and EBP and current use of health research literature. It was found that the majority of the respondents had not attended a literature appraisal skills workshop (87.3%) or received formal training in research methods (69.9%), epidemiology (91.2%) or statistics (80.8%). However, 58 (31%) stated that they had actually been personally involved in conducting some research activity. Questions regarding the participants' access to medical literature and evidence showed that only 60 (32.1%) had access to a medical library. The majority of respondents stated that they did not search for medical literature on a regular basis (86.1%), and only 75 (39.9%) respondents reported that they read every week regularly to keep up to date with their professional literature.

Table 1 illustrates computer usage by allied health professionals and CAM practitioners. Far more CAM practitioners compared to allied health professionals accessed educational literature via the Internet (95.3% v 68.1%, p = 0.008), used email on a regular basis (90.8% v 55.4%, p = 0.001) and explored and searched the Internet for information more than once a week (93.8% v 67.2%, p = 0.001 and 98% v 86.6%, p = 0.001 respectively). However, both allied health professionals and CAM practitioners identified that a vast majority of them did not have access to a library or used email discussion groups in their work.

**Table 1:** Background of allied health care professionals and complementary health practitioners concerning computer usage.

	**Total**	**n**	**%(n/t)**	**P value**
Access to the literature via Internet				
Allied health professionals	113	77	68.1	0.008
Complementary health practitioners	64	61	95.3	
				
Access to a library				
Allied health professionals	115	42	36.5	0.015
Complementary health practitioners	61	12	19.7	
				
Use of email 3 or more times a week				
Allied health professionals	121	67	55.4	
Complementary health practitioners	65	59	90.8	0.001
				
Use of email discussion groups				
Allied health professionals	111	14	12.6	0.006
Complementary health practitioners	63	19	30.2	
				
Surf the Internet more than once a week				
Allied health professionals	110	74	67.2	0.001
Complementary health practitioners	65	61	93.8	
				
Search the Internet for information more than once a week				
Allied health professionals	112	97	86.6	0.001
Complementary health practitioners	65	64	98	

**Total **= the total number of complete responses

**n **= the number of positive responses

Data missing from some respondents for each item due to incomplete responses

Only significant differences reported.

Respondents generally indicated that the majority of them did not feel confident at assessing research study design, generalisability, evaluating bias, sample size and statistical tests. Furthermore, 67.1% of practitioners felt that they had not had good or adequate training in EBM and 86.7%identified that they needed more training and education in the principles of EBM (Some confusion regarding the relationship between EBM and the process of clinical decision-making was found, with many practitioners feeling uncertain whether or not their own clinical judgement and patient choice should override the evidence. However, the majority of the practitioners agreed that they felt that EBM was essential to their practice (75.6%) and not a passing fashion (74.6%).

Respondents who reported that they had not previously attended a literature appraisal workshop stated that they felt that they needed more training in EBM compared to those that had (91.4% v 72.7%, p = 0.007), but felt that EBM has little impact on their clinical practice (26.4% v 50%, p = 0.33). The respondents who reported that they had not been involved in conducting any type of research also felt that they needed more training in EBM than those who had been involved in any research activity. However, the result was not statistically significant (91.2% v 84.5%, p = 0.09).

Table 2 examines the effect of years since qualification on practitioners' beliefs relating to EBM. We used the threshold of 11 years and over as it is unlikely that CAM practitioners who have been qualified for over ten years would have received teaching in EBP as part of their initial training. Practitioners with more than 11 years experience stated that they had not had good training previously in EBM (p = 0.04) and they felt that original research papers were confusing (p = 0.02) more often than their less experienced counterparts. The more experienced practitioners also felt that clinical judgment was more important than EBM (p = 0.005) than those with shorter length of time since qualification. More allied health care professionals stated that EBM was essential for their clinical practice and also that they needed more training in EBM than CAM practitioners (p = 0.02 and p = 0.05 respectively).

**Table 2:** Effect of profession and years since qualification (<5 years, 6–10 years, 11 years and over) on allied health care professionals and complementary health practitioners' beliefs relating to Evidence-based Medicine (EBM)

**Question**	**Total**	**Disagree (strongly to slightly) with the statement n (%)**	**Agree (strongly to slightly) with the statement n (%)**	**P value**
**Years since qualification**				
				
I find original work confusing				
5 years and under qualified	37	21 (56.7)	13 (35.1)	
6 – 10 years qualified	33	21 (63.6)	1 (3)	0.02
11 years and over qualified	66	30 (45.4)	30 (45.4)	
**OVERALL**	**136**	**72 (52.9)**	**44 (32.3)**	
				
Clinical judgement more important				
5 years and under qualified	39	18 (46.1)	21 (53.8)	
6 – 10 years qualified	36	11 (30.5)	21 (58.3)	0.005
11 years and over qualified	75	33 (44)	36 (48)	
**OVERALL**	**150**	**62 (41.3)**	**78 (52)**	
				
I had good EBM training				
5 years and under qualified	39	27 (69.2)	12 (30.7)	
6 – 10 years qualified	34	20 (58.8)	11 (32.3)	0.04
11 years and over qualified	76	53 (69.7)	15 (19.7)	
**OVERALL**	**149**	**100 (67.1)**	**38 (25.5)**	
				
**Profession**				
				
EBM is essential for my practice				
Allied health professionals	112	16 (14.2)	91 (81.2)	0.02
Complementary health practitioners	60	19 (31.6)	39 (65)	
**OVERALL**	**172**	**35 (20.3)**	**130 (75.6)**	
				
I need more training in EBM				
Allied health professionals	114	12 (10.5)	98 (85.9)	0.05
Complementary health practitioners	66	8 (12.9)	58 (93.5)	
**OVERALL**	**180**	**20 (11.1)**	**156 (86.7)**	

**Total **= total number of respondents with complete responses

**n **= number of responses

**% = **percentage of total with complete responses

Data missing from some respondents for each item due to incomplete responses

Only significant differences reported.

## Discussion and Conclusion

Our study identified several issues that require addressing in the provision of EBP training for allied health care professionals and CAM practitioners. Amongst allied and CAM practitioners the perceived need to obtain training in EBM was high and perception of competence was low. Comparatively, allied health care professionals and those with longer length of time since qualification fared worse than CAM practitioners and those with recent qualification respectively. Furthermore, learning needs varied according to the type of profession, time since graduation and prior research experience.

To ensure the validity and generalisability of our findings, we selected questions from reliable and previously validated questionnaires [18,19]. One of the strengths of our study is that we surveyed a large sample of both allied health care professionals and CAM practitioners from a variety of professions and with varying lengths of time since qualification. However, we did not employ a random sampling process and the sample was from participants who voluntarily attended the courses. Therefore our sample was restricted to those individuals who may have been more aware and self-motivated than other practitioners. Furthermore, our respondents were all based within the West Midlands region and as such our findings may not entirely reflect the knowledge and beliefs of other allied health care professionals and CAM practitioners outside of the region. We, therefore, acknowledge that the generalisability of our findings may be limited but our study does provide a starting point for further research in these groups of practitioners. However, we feel that our findings do merit consideration by teachers and trainers in these health care fields and in particular those providing continuing professional development training programmes.

Our survey suggests that like previous studies [18,19,23] that found that medical doctors lack methodological competence in critical appraisal skills and EBM, CAM practitioners and allied health care professionals also need skills. Our survey also correlates with the findings of a previous study [19] where we examined medical doctors' knowledge and beliefs concerning EBM. We found that doctors within our deanery also reported that they did not feel confident at assessing study design, generalisability of the research or evaluating sample size and statistical tests. Furthermore, many junior doctors stated that they support the principles of EBM, but they are undecided regarding whether patient choice and their own clinical judgment are more important and should override research evidence. The doctors were also in agreement with the allied health care professionals and CAM practitioners and confirmed that EBM was essential to their practice but they felt that they required further training in the subject. It is therefore, apparent that both groups have identified that EBP training is important and has not previously met their needs.

Our survey findings are exploratory and will benefit from further replication, but it does provide information for allied health care professionals' and CAM practitioners' teachers and trainers. In particular, our findings should be taken into consideration when planning EBP curricula as it is important to tailor teaching to the needs of specific subgroups of trainees to ensure that specific learning needs are met.

## Competing interests

The authors declare that they have no competing interests.

## Authors' contributions

JH contributed to the questionnaire design, co-ordination and collection of the data and drafted the manuscript. IH performed the statistical analysis and helped to draft the manuscript. KSK conceived the study, and participated in its design and contributed to the write up of the manuscript. All authors read and approved the final manuscript.

## Pre-publication history

The pre-publication history for this paper can be accessed here:



## Supplementary Material

Additional file 1 Copy of developed questionnaire. A copy of the developed questionnaire used to measure allied health care professionals and CAM practitioners' basic knowledge, skills and beliefs concerning the main principles of EBP. Click here for file
