# Supplementary material for: Knowledge and beliefs concerning evidence-based practice amongst complementary and alternative medicine health care practitioners and allied health care professionals: A questionnaire survey
Source: BMC Complement Altern Med. 2008 Jul 23;8:45. doi: 10.1186/1472-6882-8-45 (PMC2533291; doi:10.1186/1472-6882-8-45)
Supplement: Additional file 1 — Copy of developed questionnaire. A copy of the developed questionnaire used to measure allied health care professionals and CAM practitioners' basic knowledge, skills and beliefs concerning the main principles of EBP. [file 1472-6882-8-45-S1.doc]

#

# Critical Appraisal of Medical Literature & Evidence-based Medicine

# PARTICIPANTS’ KNOWLEDGE & Needs ASsESSMENT DETAILED TRAINING NEEDS ANALYSIS FORM

#

###### DETAILED TRAINING NEEDS ANALYSIS

Please take a few minutes to answer the questions below. The information you provide will be used to plan the setting up of the seminars. The aim of the questionnaire is to find out more about your experiences with computer use and medical literature appraisal.

Thank you for your participation. Your responses will be treated anonymously and confidentially.

Please tick the appropriate box(es):

1. **Your background**

1.1. Your gender *male* 

*female* 

1.2. Profession …………………………………………………….

1.3. Your professional qualifications ……………………………………………………..

1.4. Length of time since qualification / experience ……………………………………………………..

1.5. Have you ever attended a literature appraisal skills workshop? *Yes*  

*No* 

1.6. If *yes*; how long ago ………………………………………………………

1.7. Have you received any formal education or training

(e.g. degree course, seminar, workshop) in any of the following? *Please tick all that apply*

1.9.1 Research methods 

1.9.2 Epidemiology  1.9.3 Statistics 

1.8. Have you personally been involved in conducting any kind of research? *Yes* 

*No* 

# 2. Medical literature appraisal and Evidence Based Medicine (EBM)

| Please tell us briefly what you understand by the term Evidence Based Medicine (EBM)  ……………………………………………………………………………………………………………………………  ……………………………………………………………………………………………………………………………  ……………………………………………………………………………………………………………………………  …………………………………………………………………………………………………………………………… |
| --- |

1. **Access to medical knowledge**

3.1. Do you have access to a medical/healthcare library? *Yes* 

*No* 

3.2. Do you have access to literature via the Internet? *Yes* 

*No* 

- 1. Have you searched the literature for any guidelines or other

form of published evidence in the last six months? *Yes* 

*No* 

1. **Collection and dissemination of evidence**

4.1. How often, on average do you search for evidence? *Please tick the appropriate box*

4.1.1. More than once a week 

4.1.2. Every 1-2 weeks 

4.1.3. Every 3-4 weeks 

4.1.4. Less than once a month 

4.1.5. Never 

4.2. Do you keep up to date with your professional literature? *Please tick the appropriate box*

4.2.1. Yes – read every week regularly 

4.2.2. Yes – read occasionally 

4.2.3. Yes – only for specific information 

4.2.4. No 

4.3. What type of material do you read to find information about evidence for your practice?

Please tick all that apply

4.3.1. Journals: review articles 

4.3.2. Journals: original research reports 

4.3.3. Textbooks 

4.3.4. Internet resources or similar 

4.3.5. Hospital guidelines 

4.3.6. Hospital Intranet guidelines 

4.3.7. The Cochrane library 

4.3.8. Nice guidelines 

4.3.9. Clinical guidelines (BMJ publication) 

4.4.0. Evidence based medicine journal 

4.4.1. Other 

# 5. Computer use

How often have you personally…?

| - 1. Written a paper (longer than five pages) using a word processing program (e.g. Microsoft Word) |  Never  1–2 times  3 or more times |
| --- | --- |
| - 1. Sent or received an electronic mail (e-mail) message |  Never  1–2 times  3 or more times |
| - 1. Participated in an email discussion group |  Never  1–2 times  3 or more times |
| - 1. Chatted using windows messenger? |  Never  1–2 times  3 or more times |
| - 1. Used a computer-assisted instruction (CAI) program, possibly as a college student |  Never  1–2 times  3 or more times |
| - 1. Taken a class online |  Never  1–2 times  3 or more times |
| - 1. Explored the Internet using Netscape, Internet Explorer or other |  Never  1–2 times  3 or more times |
| - 1. Used the Web to search for information |  Never  1–2 times  3 or more times |
| - 1. Used any medical resources on the Internet (e.g MEDLINE, MICROMEDEX) |  Never  1–2 times  3 or more times |
| 5.10. Used any medical diagnosis and decision- support software (lliad, QMR, etc) |  Never  1–2 times  3 or more times |
| 5.11. Do you own a personal computer? | *Yes*   *No*  |
| 5.12. If *yes* which of the following does it have? | *please tick all that apply*  CD-ROM drive   Modem   Video card   Internet service provider (ISP) or online service  |
| 5.13. Do you have access to a computer room with Internet and network facilities? | *Yes*   *No*   If *yes* where?………………………………………….. |
| 5.14. Do you have your own Web page? | *Yes*   *No*  |

**6. How confident do you think you are at assessing each of these aspects of a published paper?**

*Please circle one for each that best represents your level of confidence. Please indicate if you do not understand the question.*

*These are all on a 1-6 scale.*

**1** meaning ***no confidence at all* 2** means ***not very confident* 3** means ***slightly not confident***

**4** means ***slightly confident* 5** means ***confident* 6** means ***very confident***

| **Not Very**  **Confident Confident**  At all     - 1. Assessing study design 1 2 3 4 5 6 | ***If you do not understand the question please tick below***   |
| --- | --- |
| - 1. Evaluating bias 1 2 3 4 5 6 |  |
| - 1. Evaluating the 1 2 3 4 5 6   adequacy of sample size |  |
| - 1. Assessing generalisability 1 2 3 4 5 6 |  |
| - 1. Evaluating statistical tests 1 2 3 4 5 6   /principles |  |
| - 1. Assessing the general worth 1 2 3 4 5 6   of an article |  |

**7. What are your beliefs about evidence based medicine (EBM)?**

*Instructions:*

*Below are ten statements about literature appraisal and evidence based medicine (EBM)*

*Please read each statement carefully and then circle the number, which reflects your views most closely. Please indicate if you do not understand the question.*

These are all on a 1-6 scale.

**1** meaning ***strongly disagree* 2** means ***disagree* 3** means ***slightly disagree***

**4** means ***slightly agree* 5** means ***agree* 6** means ***strongly agree***

| **Disagree Agree**  **Strongly strongly** | ***If you do not understand the question please tick below*** |
| --- | --- |
| 7.1. I think that original research is 1 2 3 4 5 6  confusing |  |
| 7.2. EBM is essential in my 1 2 3 4 5 6  practice |  |
| 7.3. I feel I need more training in EBM 1 2 3 4 5 6 |  |
| 7.4. I am confident I can assess 1 2 3 4 5 6  research evidence |  |
| 7.5. Systematic reviews are key to 1 2 3 4 5 6  informing EBM |  |
| 7.6. EBM has little impact on an 1 2 3 4 5 6  individual’s practice |  |
| 7.7. I have received a lot of good 1 2 3 4 5 6  training in EBM |  |
| 7.8. Clinical judgement is more 1 2 3 4 5 6  important than EBM |  |
| 7.9. Patient choice should override 1 2 3 4 5 6  EBM |  |
| 7.10. EBM is a passing fashion 1 2 3 4 5 6 |  |

### Please feel free to add any further comments you wish in this box
